# Reduction of Mycotoxigenic Fungi Growth and Their Mycotoxin Production by *Bacillus subtilis* QST 713

**DOI:** 10.3390/toxins14110797

**Published:** 2022-11-17

**Authors:** Terenzio Bertuzzi, Giulia Leni, Giulia Bulla, Paola Giorni

**Affiliations:** 1 Department of Animal Science, Food and Nutrition, Università Cattolica del Sacro Cuore, 29122 Piacenza, Italy; 2 Department of Sustainable Crop Production, Università Cattolica del Sacro Cuore, 29122 Piacenza, Italy

**Keywords:** *Bacillus subtilis* QST 713, mycotoxin, mycotoxigenic fungi, lipopeptides

## Abstract

The use of chemical pesticides to control the occurrence of mycotoxigenic fungi in crops has led to environmental and human health issues, driving the agriculture sector to a more sustainable system. Biocontrol agents such as *Bacillus* strains and their antimicrobial metabolites have been proposed as alternatives to chemical pesticides. In the present work, a broth obtained from a commercial product containing *Bacillus subtilis* QST 713 was tested for its ability to inhibit the growth of mycotoxigenic fungi as well as reduce their mycotoxin production. Mass spectrometry analysis of *Bacillus subtilis* broth allowed to detect the presence of 14 different lipopeptides, belonging to the iturin, fengycin, and surfactin families, already known for their antifungal properties. *Bacillus subtilis* broth demonstrated to be a useful tool to inhibit the growth of some of the most important mycotoxigenic fungi such as *Aspergillus flavus*, *Fusarium verticillioides*, *Fusarium graminearum*, *Aspergillus carbonarius*, and *Alternaria alternata*. In addition, cell-free *Bacillus subtilis* broth provided the most promising results against the growth of *Fusarium graminearum* and *Alternaria alternata*, where the radial growth was reduced up to 86% with respect to the untreated test. With regard to the mycotoxin reduction, raw *Bacillus subtilis* broth completely inhibited the production of aflatoxin B1, deoxynivalenol, zearalenone, and tenuazonic acid. Cell-free broth provided promising inhibitory properties toward all of the target mycotoxins, even if the results were less promising than the corresponding raw broth. In conclusion, this work showed that a commercial *Bacillus subtilis*, characterized by the presence of different lipopeptides, was able to reduce the growth of the main mycotoxigenic fungi and inhibit the production of related mycotoxins.

## 1. Introduction

**Figure 1:**
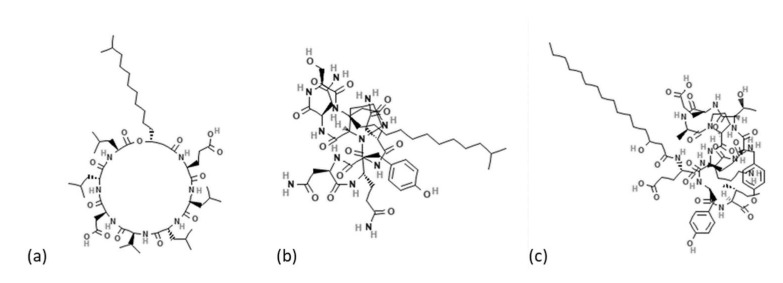
(**a**) Surfactin, (**b**) iturin, and (**c**) fengycin structures.

Mycotoxigenic fungal contamination represents a serious threat to crop plant production and global food security. In the past years, biofungicides have been proposed as a sustainable management system in order to reduce and substitute the use of chemical pesticides to shift the agriculture sector to greener approaches [1]. Bacteria, and in particular bacilli, are considered as one of the most studied groups of biofungicides, able to provide plant growth-promotion and the biocontrol of multiple diseases [2]. The antagonistic effect of *Bacillus* species is mainly related to the production of various bioactive secondary metabolites and lytic enzymes [3]. Lipopeptides, cyclic, low molecular weight, and amphiphilic molecules with hydrophilic and hydrophobic moieties have been proposed as one of the strongest antimicrobial and antifungal secondary metabolites produced by several species of the genus *Bacillus* [4]. Surfactins, iturins, and fengycins are the three most well-known families of lipopeptides. Surfactins and iturins consist of a ring of seven α-amino acids linked to one β-hydroxy (surfactins) or β-amino (iturins) fatty acid; fengycins are formed by a ring of eight α-amino acids linked to ornithine and glutamic acid and to one β-hydroxy fatty acid (Figure 1).

The length of fatty-acid chains varies from 11 to 17 C atoms for surfactins [5,6], 14 to 17 C for iturins, and from 14 to 19 C for fengycins [7]. Generally, iturins and fengycins show strong antifungal activities, while surfactins have an antibacterial activity; these compounds show a very low toxicity for humans, high biodegradation, and can be applied in both pharmaceutical, food, and agriculture research [8]. Lipopeptides producing *Bacillus* species are often endophytes for different agriculture crops, producing positive effects on the host [9,10]. They exert their activity as an elicitor of biochemical changes (e.g., reinforcements of plant cell walls, production of antimicrobial phytoalexins, and the synthesis of pathogenesis-related enzymes) that trigger plant induced systemic resistance [11]. In addition, several studies have shown encouraging results for their antifungal properties in vitro or in vivo on different crops. Lipopeptides have been demonstrated to directly act on fungi by inhibiting spore germination, germ tubes, and hyphal growth of filamentous fungi [12], even if the specific mechanisms behind these antimicrobial bioactivities remain largely unknown [13]. In common bean seeds, *Bacillus amyloliquefaciens* prevented the development of endophytic fungi [14]; lipopeptides produced by endophytic *Bacillus subtilis* induced host defense gene expression in maize [15]. Moreover, fengycin and iturin compounds inhibited different phytopathogenic fungi of wheat [16]. Most recently, different studies have investigated the effects of lipopeptides on the main mycotoxigenic fungi and their mycotoxin production [11,17]. Antifungal activity against the aflatoxin-producing fungi *Aspergillus flavus* and *Aspergillus parasiticus* was shown by iturin produced by *Bacillus pumilus* isolated from soybean sauce [18]; lipopeptides produced by *Bacillus vallismortis* were successful tested against *Alternata alternata* [17]. Their use as natural compounds produced by microbial populations was proposed in combination with synthetic fungicides and may be a safe and promising alternative [19]; Kihyun Kim indicated that lipopeptides produced by *Bacillus amyloliquefaciens* could reduce the growth of *Fusarium graminearum* and trichothecene production and could be used as chemosensitizers to chemical fungicides [20]. 

Nowadays, *Bacillus*-based products, also engineered to improve lipopeptide yield [21], are commercially available as biocontrol agents against phytopathogenic fungi and are employed directly to the soil or sprayed in the plant surface [22,23]. In the present work, a commercial *Bacillus subtilis* QST 713 was studied for its ability to produce different lipopeptides and inhibit the presence of some of the main mycotoxigenic fungi as well as their regulated mycotoxins. For this purpose, in vitro tests were carried out in order to verify the reduction in aflatoxins, fumonisins, deoxynivalenol, ochratoxin A, zearalenone, and *Alternaria* toxins.

## 2. Results and Discussion

### 2.1. Lipopeptide Production of Bacillus subtilis QST 713

Raw and cell-free *Bacillus subtilis* broths were analyzed for lipopeptide determination, as reported in the Materials and Methods section and in Table 1. 

Regarding surfactins, an average concentration of 292 mg/L for surfactin C was determined in the raw broth. Other surfactins with MW 1023, 1008, and 994 Da (probably C14, C13, and C12 fatty acid chain) showed lower signals of 72.6, 8.9, and 2.2% with respect to that of surfactin C. In the cell-free broth, the concentration of surfactins were always lower, with percentages of reduction that ranged from 5% for the C12 surfactin to 30% for the C15 surfactin with respect to the signals detected in raw broth. Regarding the C13 and C14 surfactins, they presented signals that were 25.3% and 27.5% lower than those detected in raw broth, respectively.

For the iturins, the average concentration of iturin A in the raw broth was 3034 mg/L; the highest peak area was represented by the isoform A, C14 fatty acid chain-iturin with a MW of 1043 Da; the signals of iturins with 1057, 1071, and 1085 Da (probably C15, C16 and C17) were 40.3, 24.1, and 5.4%, respectively, to that of iturin A. In the cell-free broth, as also observed for surfactin compounds, it was detected a lower concentration of iturin isoforms. The percentages of reduction were 6.9 % for C14 iturin, 23.9% for the C15 iturin, 26.1% for the C16 iturin and 44.1% for the C17 iturin compared to their respective signals detected in the raw broth. 

Finally, with regard to the fengycin family, the ones with a MW of 1464 Da (C16 fatty acid chain, called fengycin C) and 1492 Da (C18) showed the greatest signals. An average concentration value of 1033 mg/L of fengycin C was calculated in the raw broth. For fengycin with 1478 and 1506 Da, the signals were 52.3 and 29.0%, respectively, compared to that of fengycin C; fengycins with a MW of 1436 and 1450 Da were detected at very low levels (3.8 and 5.1% to fengycin C, respectively). Limited reductions ranging from 1.0 to 16.7% were observed in the cell-free broth compared to the lipopeptide signals detected in the raw broth. In particular, these percentage reductions were about 1.0% for C15 fengycin, 2.5% for C16 fengycin, 6.5% for C14 fengycin, 8.6% for C18 fengycin, 13.1 and 16.7 % for C17 and C19 fengycin, respectively.

### 2.2. Reduction on Fungal Growth and Mycotoxin Production

Raw and cell-free *Bacillus subtilis* broths were tested for their in vitro ability to reduce the growth of mycotoxigenic fungi and mycotoxin production. In the case of raw *Bacillus subtilis* broth, where bacterial cells were still present and vital, the growth of all of the tested mycotoxigenic fungi resulted in being completely inhibited. Using the cell-free broth, instead, the presence of the bacteria was completely avoided, and the fungal growth was presumably limited by the composition of the bacterial metabolites present. In particular, the best results were obtained against *Fusarium graminearum* and *Alternaria alternata* with a reduction in growth higher than 85%. The lowest incidence of fungal growth was obtained by *Aspergillus flavus*, where no significant differences were determined between the untreated control and the *Bacillus subtilis* cell-free broth (Table 2).

Regarding mycotoxins, the raw *Bacillus subtilis* broth completely inhibited the production of aflatoxin B1, deoxynivalenol, zearalenone, and tenuazonic acid and an average reduction between 93.1 and 99.7% was obtained for ochratoxin A, fumonisin B1 and B2, alternariol, alternariol mono-ether, and tentoxin (Table 3). 

**Figure 2:**
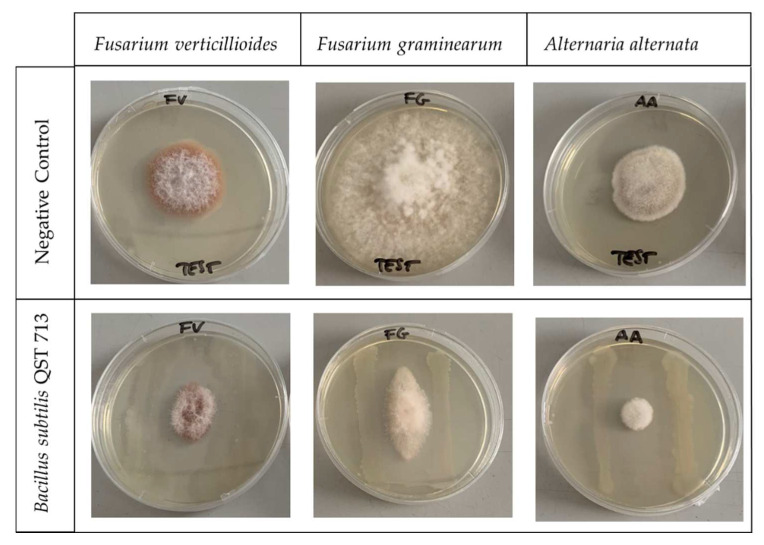
Dual culture plate assay of *Bacillus subtilis* QST 713 antagonistic effect against *Alternaria alternata, Fusarium graminearum* and *Fusarium verticilloides*. The negative control assay was performed in the same condition but without the *Bacillus subtilis* strip. Petri dishes were incubated at 25 °C for 14 days.

The *Bacillus subtilis* cell-free broth showed results on mycotoxin inhibition less promising that the raw one, as also previously reported for the lipopeptide concentration and the fungal growth. The lower inhibitory capacity of cell-free broth was probably due to the lower concentration of *Bacillus subtilis* secondary metabolites with antifungal activities and to the continuous production of lipopeptides by the bacterium in the non-filtered broth. The inhibitory activity of these lipopeptides has been demonstrated by a dual plate trial performed by positioning the fungal inoculum at the center of a Petri dish with a bacterial strip on both side of the fungus (Figure 2).

None or very limited fungal growth was observed, and no contact took place between the fungus and the bacterium strip. In addition, in the portion of agar free of any visible contamination, the presence of lipopeptides was detected, underlining that these compounds were secreted by *Bacillus subtilis* and surely contributed to the inhibition of fungal growth with other undetected antimicrobial metabolites.

Biocontrol agents based on *Bacillus* species and the derived lipopeptides are nowadays available on the market [24,25]. Their activity ranged among a large spectrum of crop diseases: grey mold (*Botrytis cinerea*); scab (*Venturia* spp.); fire blight (*Erwinia amylovora*); *Sclerotinia* spp.; monilia (*Monilia fructigena*); bacterial spot of stone fruits (*Xanthomonas arboricola*); and bacterial speck in tomato plants (*Pseudomonas syringae* pv. tomato). In the scientific literature, several studies reported the inhibitory activity of lipopeptides against the growth of some mycotoxigenic fungi [26,27,28]. A reduction in fungal growth does not imply a minor mycotoxin contamination; in fact, under stress conditions, fungi can indeed increase mycotoxin production [29,30]. In this direction, recent studies have also demonstrated the high efficacy of these lipopeptides on the reduction of some mycotoxins. In particular, fengycins, purified from the *Bacillus amyloliquefaciens* extract, were able to suppress *Fusarium graminearum* growth and reduce deoxynivalenol, 3-acetyldeoxynivalenol, 15-acetyldeoxynivalenol, and zearalenone production in infected grains [31]; iturin A significantly inhibited the growth and production of ochratoxin A and *Aspergillus carbonarius* [32]. A mixture of surfactins and fengycins produced by *Bacillus mojavensis* suppressed the growth of *Fusarium oxysporum* and inhibited T-2 and HT-2 toxin production [33]. Fengycin inhibited patulin production and the gene expression of patulin in *Penicillium expansum* [34]. Finally, next to lipopeptides, *Bacillus* strains have been characterized for their ability to produce a wide spectrum of different secondary metabolites including enzymes (e.g., chitinases, glucanases, and proteases) that can exert antifungal activities [35] and have not been characterized here.

## 3. Conclusions

*Bacillus* species have been reported to produce a plethora of secondary metabolites with excellent biocontrol properties, with lipopeptides that display strong antifungal activity [36]. In this study, it was demonstrated by in vitro assays that a commercial *Bacillus subtilis* QST 713 is able to suppress the growth of the main mycotoxigenic fungi and to inhibit the production of related mycotoxins. In addition, the *Bacillus subtilis* QST 713 broths have been characterized by mass-spectrometry analysis for the presence of different lipopeptides belonging to fengycin, surfactin, and iturin families. It is not excluded that the *Bacillus subtilis* QST 713 is able to produce other antifungal metabolites such as enzymes, which could have co-participated with lipopeptides in the inhibition of fungal growth and mycotoxin production. In this direction, further studies are needed in order to confirm the presence of other antifungal compounds and identify the ones most affective against the different mycotoxigenic fungi tested here. In addition, in field analysis on different crops (i.e., cereals, horticultural products) will be needed in order to confirm these preliminary data. In this way, it will be possible to substitute or reduce chemical pesticides, which are nowadays used for controlling the occurrence of mycotoxigenic fungi, with more sustainable alternatives that are already used for other crop diseases. The decrease in synthetic fungicide use will accelerate the transition to a more sustainable agriculture system, perfectly meeting the Farm to Fork strategy proposed by the European Union Green Deal.

## 4. Materials and Methods

### 4.1. Reagents and Standards

The chemicals and solvents used for the extraction and clean-up solutions were ACS grade or equivalent (Carlo Erba, Milano, Italy). Deionized water was purified through a Milli-Q treatment system (Millipore, Bedford, MA, USA). For LC-MS/MS analysis, water, methanol, acetonitrile, and formic acid were of HPLC grade (Merck, Darmstadt, Germany). Toxins (AFB1, OTA, FB1, FB2, DON, ZEA, AOH, AME, TEA, and TEN) and lipopeptide standards (surfactin C, iturin A, fengycin C) were obtained from Sigma-Aldrich (St. Louis, MO, USA). 

### 4.2. Preparation of Fungal Strains 

One strain of *Aspergillus flavus* (ITEM 8069), one strain of *Fusarium verticillioides* (ITEM 10027), one strain of *Fusarium graminearum* (ITEM 646), one strain of *Aspergillus carbonarius* (ITEM 5012) from the official fungal collection of the Institute of Sciences of Food Production of the National Research Council (ISPA-CNR) in Bari (Italy) and one strain of *Alternaria alternata* (CBS 118814) from the Westerdijk Fungal Biodiversity Institute in Utrecht (The Netherlands) were used in this work. The fungal strains were transferred in the center of Petri dishes containing potato dextrose agar (PDA, Biolife, Milano, Italy) and incubated at 25 °C for 7 days (12 h light + 12 h dark photoperiod) [37,38,39]. After the incubation period, the developed fungal colonies were used as the source of inoculum for the in vitro test.

### 4.3. Preparation of Bacterial Solution

One gram of a commercial product based on *Bacillus subtilis* QST 713 was added to 1000 mL of potato dextrose broth (PDB) obtained from potato broth (200 g of potatoes/L of distilled water, 10g/L of D-glucose); the solution was incubated at 25 °C for 3 weeks before being used in the in vitro experiments to evaluate its potential to reduce fungal growth both prior and after filtration with a sterile 0.20 µm filter in order to obtain a cell-free *Bacillus subtilis* broth.

### 4.4. Lipopeptide Analysis

After dilution (1 mL of bacterial solution + 4 mL of mixture H_2_O:CH_3_CN = 80:20) and filtration (0.45 µm), the determination of lipopeptides was performed by LC-MS/MS. Filtration was necessary to eliminate any kind of solid in the broths before injection in the instrument. However, after this operation, even bacterial cells present in row *B. subtilis* broth were removed. The system consisted of a Vanquish pump and autosampler, and a TSQ Fortis triple-quadrupole mass spectrometer (Thermo-Fisher Scientific, San Jose, CA, USA). The separation was performed with a Betasil RP-18 column (5 µm particle size, 150 mm × 2.1 mm, Thermo-Fisher) with a gradient H_2_O:CH_3_CN (both acidified with 0.2% of formic acid) from 75:25 to 5:95 in 5 min, isocratic for 10 min; the flow rate was 0.2 mL/min and the injection volume 10 µL. Ionization was carried out in positive and detection in selected ion monitoring mode; a total of four surfactins, four iturins, and six fengycins were selected (Table 1). Standards of surfactin C (MW 1036 Da), iturin A (MW 1043 Da), and fengycin C (MW 1464 Da, Sigma-Aldrich, Milano, Italy) were injected.

### 4.5. In Vitro Experiment

Considering both the raw and cell-free *Bacillus subtilis* broths, 1 mL of the solution was added on Petri dishes containing PDA medium and distributed on the surface with a sterile spatula. Agar discs (Ø 2mm) were cut with a sterile cork borer from the margin of one of the mycotoxigenic fungal colony and put at the center of the dish. An untreated thesis (Petri dishes without the addition of the bacterial broths) was also tested and considered as the control for all five fungal strains. Then, Petri dishes were incubated at 25 °C for 14 days and, at the end of the incubation time, the fungal growth and mycotoxin production were determined. The test was conducted in triplicate. 

### 4.6. In Vitro Bacterial Effect on Fungal Growth

The diameter of the fungal colonies was measured along two perpendicular diagonals crossing the inoculum point. The percentage of reduction in fungal growth was calculated by comparing the fungal growth diameters obtained in the untreated dishes (Petri dishes without the bacterial broths) with those obtained in the presence of the bacterial broths.

### 4.7. Mycotoxin Analysis

The mycotoxins were extracted and analyzed as reported by methods previously developed in our laboratory. Briefly, the fungal colony and agar media were mixed in a flask with 40 mL of acetonitrile and vigorously shaken with a rotary-shaking stirrer for 1 h. The mixture was then filtered and diluted for instrumental analysis. For fumonisins and *Alternaria* toxins, the analysis was carried out by LC-MS/MS, according to the methods of Pietri and Bertuzzi [15] and Bertuzzi et al. [16], respectively. Briefly, both groups of toxins were separated on a Betasil RP-18 column (5 μm particle size, 150 mm × 2.1 mm, Thermo-Fisher, Milano, Italy). For fumonisins, a mobile-phase gradient acetonitrile–water (both acidified with 0.2% formic acid) from 25:75 to 55:45 in 9 min was performed, then isocratic for 3 min; the flow rate was 0.2 mL min^−1^. *Alternaria* toxins were separated using gradient elution with acetonitrile and water (both acidified with 0.2% formic acid) from 35:65 to 75:25 in 5 min, then isocratic for 2 min, at a flow rate of 0.2 mL min^−1^. For the fragmentation of [M + H]+ ions (722 *m*/*z* for FB1, 706 *m*/*z* for FB2, 259 *m/z* for AOH, 273 *m*/*z* for AME, 198 *m*/*z* for TeA and 415 *m*/*z* for TEN), the fragment ions were: 704, 352, and 334 *m*/*z* for FB1; 688, 336, and 318 *m*/*z* for FB2; 738, 374, 128, 185, and 213 *m*/*z* (35 V) for AOH, 128, 184 *m*/*z* (38 V), and 258 *m*/*z* (30 V) for AME, 125, 139, and 153 *m*/*z* (16 V) for TeA, 132 *m*/*z* (37 V), 135, and 312 *m*/*z* (25 V) for TEN. Deoxynivalenol and zearalenone were detected by GC-MS and HPLC-FD as described by Bertuzzi et al. [17]. Briefly, GC-MS analysis was carried out using a TraceGQ Ultra coupled with an ISQ single quadrupole mass spectrometry (Thermo-Fisher Scientific, Milano, Italy). A capillary column Rtx-5MS (30 m × 0.25 mm i.d., 0.25 µm film thickness; Restek Corporation, Bellefonte, PA, USA) was used for the analysis. Helium was the carrier gas with a column head pressure of 55 kPa. The PTV temperature was raised from 70 °C (held 0.2 min) to 260 °C (held for 2 min) at 10 °C sec^−1^. The oven temperature programming was from 125 °C (held for 1 min) to 245 °C at 10 °C min^−1^ and then to 300 °C (held for 1 min) at 30 °C min^−1^. MS transfer line and ion source temperature were at 230 °C and 250 °C, respectively. Electron ionization at 70 eV and selected ion monitoring (SIM) were used for DON detection (fragment monitored: 393, 407, 422, 512). Zearalenone was detected with an HPLC system (Perkin Elmer, Norwalk, CT, USA) equipped with a FP 1520 fluorescence detector (Jasco Corporation, Lecco, Italy) set at 274 nm excitation and 440 nm emission wavelengths. ZEN was separated on a phenyl-hexyl column (5 µm particle size, 150 mm × 4.6 mm i.d.; Phenomenex, Torrance, CA, USA) at ambient temperature with a mobile phase acetonitrile—2% acetic acid aqueous solution (43 + 57 *v*/*v*) at 1.0 mL min^−1^. Aflatoxins and ochratoxin A were determined as reported by Pietri et al. [18]. The analysis was performed with a HPLC instrument equipped with a FP 1520 fluorescence detector (Jasco Corporation, Tokyo, Japan). AFs were separated with a Superspher RP-18 column (4 μm particle size, 125 mm × 4 mm i.d., Merck) while OTA with a phenyl-hexyl column (5 μm particle size, 150 mm × 4.6 mm i.d., Phenomenex, Torrance, CA, USA) at ambient temperature. For AFs, the mobile phase was water–methanol–acetonitrile (64 + 23 + 13, *v*/*v*/*v*), the flow rate was 0.5 mL min^−1^, and the fluorimeter was set at 365 nm excitation and 440 nm emission wavelengths. OTA were analyzed with a mobile phase gradient acetonitrile–2% acetic acid aqueous solution from 35:65 to 67:33 in 15 min, the flow rate was 1.0 mL min^−1^, the fluorescence detector was set at 333 nm excitation and 470 nm emission wavelengths.

### 4.8. Data Analysis 

The data were transformed before statistical analysis; in particular, fungal growth reduction was arcsine transformed and mycotoxin content was ln transformed [19]. Analysis of variance (ANOVA) was calculated using the statistical package IBM SPSS Statistics 27 (IBM Corp., Armonk, NY, USA) while significant differences were highlighted using the Tukey test (*p* ≤ 0.05) for mean separation.

## Figures and Tables

**Table 1:** Lipopeptides detected in the *Bacillus subtilis* broth.

Lipopeptide Family	[M-H]^+^	Possible Assignment	Retention Time
Surfactin C	995	Surfactin C12	13.95
	1009	Surfactin C13	14.93
	1023	Surfactin C14	17.31
	1037	Surfactin C15	18.68
Iturin A	1044	Iturin C14	7.02
	1058	Iturin C15	7.25
	1072	Iturin C16	7.66
	1086	Iturin C17	7.93
Fengycin C	1437	Fengycin C14	7.19
	1451	Fengycin C15	7.67
	1465	Fengycin C16	7.70
	1479	Fengycin C17	7.86
	1493	Fengycin C18	7.89
	1507	Fengycin C19	8.06

**Table 2:** Antagonistic activity of cell-free Bacillus subtilis broth against toxigenic fungi.

Fungal Isolates	Treatments	Average Radial Fungal Growth (mm)	Average Reduction %
*Aspergillus flavus*	Control (untreated)	77.5 ± 2.5 ^a^	
	+ *Bacillus subtilis* cell-free broth	70.2 ± 16.0 ^a^	9.5
*Fusarium verticillioides*	Control (untreated)	81.0 ± 0.0 ^a^	
	+ *Bacillus subtilis* cell-free broth	59.7 ± 2.5 ^b^	26.3
*Fusarium graminearum*	Control (untreated)	85.0 ± 0.0 ^a^	
	+ *Bacillus subtilis* cell-free broth	12.2 ± 2.5 ^b^	85.7
*Aspergillus carbonarius*	Control (untreated)	73.8 ± 1.3 ^a^	
	+ *Bacillus subtilis* cell-free broth	50.5 ± 4.7 ^b^	31.5
*Alternaria alternata*	Control (untreated)	72.3 ± 3.8 ^a^	
	+ *Bacillus subtilis* cell-free broth	10.0 ± 3.3 ^b^	86.2

The data are reported as average ± standard deviation and are the mean of three independent analyses. Significant differences were compared among each fungal strain trials at a level of *p* < 0.05 and are indicated by different letters.

**Table 3:** Effect of *Bacillus subtilis* raw and cell-free broths on mycotoxins.

	*Aspergillus* *flavus*	*Aspergillus* *carbonarius*	*Fusarium* *verticillioides*		*Fusarium* *graminearum*		*Alternaria* *alternata*			
Treatment	AFB1	OTA	FB1	FB2	DON	ZEA	AOH	AME	TEA	TEN
Control (untreated)	182.9 ± 8.0 ^a^	63333 ± 13053 ^a^	752.2 ± 91.3 ^a^	182.3 ± 22.0 ^a^	100.8 ± 22.4 ^a^	109.2 ± 1.9 ^a^	323.9 ± 77.3 ^a^	1513.7 ± 235.7 ^a^	1678.0 ± 270.8 ^a^	21.3 ± 5.3 ^a^
Raw *Bacillus subtilis* broth	<0.1 ^c^	184.3 ± 146 ^c^	51.7 ± 34.5 ^c^	10.7 ± 7.4 ^b^	<0.5 ^c^	<0.2 ^c^	3.7 ± 0.3 ^c^	39.7 ± 6.8 ^b^	<0.5 ^b^	0.3 ± 0.1 ^b^
*Bacillus subtilis*-cell-free broth	53.2 ± 31.6 ^b^	2700 ± 1450 ^b^	305.7 ± 67.6 ^b^	52.3 ± 36.7 ^b^	10.5 ± 3.9 ^b^	41.8 ± 15.5 ^b^	11.5 ± 5.1 ^b^	46.1 ± 24.8 ^b^	<0.5 ^b^	1.2 ± 0.8 ^b^

Aflatoxin B1 (AFB1), ochratoxin (OTA), fumonisins B1 and B2 (FB1 and FB2), deoxynivalenol (DON), zearalenone (ZEA), alternariol (AOH), alternariol mono ether (AME), tenuazonic acid (TEA), tentoxin (TEN) production. Results are expressed as μg/g and are the mean of three independent analyses. Values followed by different letters within one column are significantly different (*p* < 0.05).

## Data Availability

Not applicable.
